# Transient Global Amnesia with Reversible White Matter Lesions: A Variant of Posterior Reversible Encephalopathy Syndrome?

**DOI:** 10.1155/2015/541328

**Published:** 2015-11-30

**Authors:** Tomoki Nakamizo, Ippei Tsuzuki, Takashi Koide

**Affiliations:** Department of Neurology, Hiratsuka City Hospital, 1-19-1 Minamihara, Hiratsuka, Kanagawa 254-0065, Japan

## Abstract

Transient global amnesia (TGA) is a self-limited disease characterized by isolated amnesia, which resolves within 24 h. In contrast, posterior reversible encephalopathy syndrome (PRES) is a potentially life-threatening disease that usually presents with seizures, altered mental status, headache, and visual disturbances. It is characterized by reversible vasogenic edema that predominantly involves the parieto-occipital subcortical white matter as shown by neuroimaging studies. To date, there have been no reported cases of PRES with a clinical course resembling TGA. Here we report the case of a 58-year-old woman who presented with isolated amnesia and headache. On admission, her blood pressure was 187/100 mmHg. She had complete anterograde amnesia and slight retrograde amnesia without other neurological findings. After the treatment of her hypertension, the amnesia resolved within 24 h. Although the initial magnetic resonance image (MRI) was almost normal, the fluid attenuation inversion recovery (FLAIR) images of the MRI on the next day revealed several small foci of high intensity areas in the fronto-parieto-occipital subcortical white matter, presumed to be vasogenic edema in PRES. The lesions disappeared one month later. This case suggests that PRES can mimic the clinical course of TGA. PRES should be considered in the differential diagnosis for TGA.

## 1. Introduction

Both transient global amnesia (TGA) and posterior reversible encephalopathy syndrome (PRES) are acute neurological disorders that are frequently encountered in emergency and neurological departments. TGA is a self-limited disease characterized by the abrupt onset of complete anterograde and mild retrograde amnesia that resolves within 24 h [1–3]. It is not accompanied by other neurological symptoms or signs; however, it is often accompanied by nonfocal symptoms, such as headache and nausea [1–3]. PRES presents with acute neurological symptoms, such as seizures, altered mental status, headache, and visual disturbances, triggered by certain factors including hypertension, cytotoxic drugs, autoimmune disorders, and preeclampsia or eclampsia [4, 5]. It is characterized by reversible vasogenic edema that predominantly involves the parieto-occipital subcortical white matter as shown by neuroimaging studies [4–6]. Although PRES can have various presentations [4], no cases of PRES with a clinical course resembling TGA have been reported. Here we present the case of a patient whose clinical course resembled TGA and whose radiological findings were consistent with PRES.

## 2. Case Presentation

A 58-year-old woman presented with acute memory disturbance and headache. During the afternoon of the day of presentation, she did not have any complaints while she was at work. After finishing work, she drove home. By the time she arrived at her home, she developed a headache and nausea. Her husband noticed that her memory was severely disturbed and called an ambulance.

In the emergency department, she complained of headache and nausea but denied any visual disturbances. Her blood pressure was 187/100 mmHg, and her pulse rate was 77 beats per min. The remainder of the physical examination was normal. Neurologically, she was alert and oriented with respect to place and person but disoriented with regard to time. She was unable to acquire new information and to recall the events that occurred during the same or previous day. She repeatedly asked the same questions. Her remote and working memories were intact, and she showed no loss of personal identity. Her speech and reasoning were intact. There was no visual field defect, and the other neurological examinations were normal.

**Figure 1:**
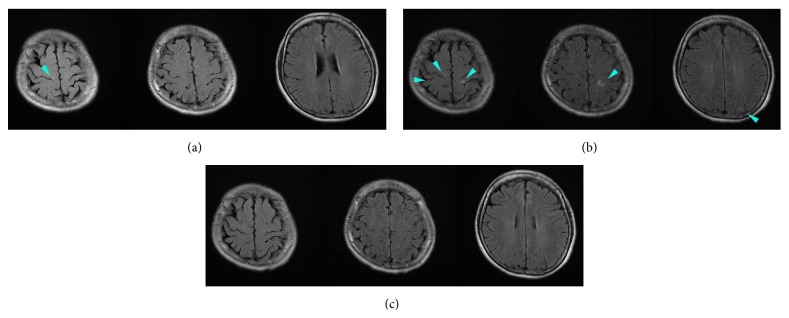
(a) Fluid attenuation inversion recovery (FLAIR) images of MRI on admission were interpreted as normal, but in retrospect, they show an indistinct high-intensity area in the right frontal subcortical white matter (arrowhead). (b) FLAIR images of MRI on the next day reveal high-intensity areas, presumed to be vasogenic edema, in the right frontal, bilateral parietal, and left occipital subcortical white matter (arrowheads). (c) The repeat MRI performed one month later shows the disappearance of all lesions.

**Figure 2:**
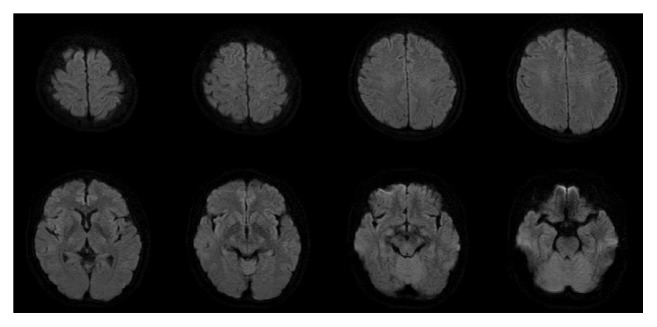
Diffusion-weighted images (DWIs) of MRI on the next day of admission were normal.

Her laboratory tests, including those of the cerebrospinal fluid, were normal, and her brain magnetic resonance image (MRI) (Figure 1(a)) was interpreted as normal. She was suspected of having a diagnosis of TGA. However, because we were concerned about her high blood pressure along with the headache and nausea, we treated her hypertension with nicardipine, observed her in the emergency ward, and planed a follow-up MRI looking for punctate high intensity spots in hippocampus on diffusion-weighted images (DWIs), the diagnostic finding for TGA. Subsequently, over the next 9 h, her blood pressure returned to normal, and her amnesia improved. She no longer had nausea. By the next morning, her amnesia had completely resolved, although there was a gap in her memory during the episode. The DWIs (Figure 2) of the brain MRI on the same day were normal, but the fluid attenuation inversion recovery (FLAIR) images (Figure 1(b)) revealed high-intensity areas in the subcortical white matter of her left occipital, bilateral parietal, and right frontal lobes. Although small, the distribution and subcortical location of these areas were characteristic of vasogenic edema of PRES. In retrospect, only the right frontal lesion was discernable in the first MRI (Figure 1(a)). The magnetic resonance angiogram was normal. The following day, her blood pressure remained normal, and her headache resolved. She was placed on antihypertensive medications and discharged without any neurological deficits. One month later, a repeat MRI (Figure 1(c)) showed that all lesions had disappeared. We finally diagnosed her with PRES.

## 3. Discussion

This patient presented with complete anterograde amnesia in the absence of other neurological symptoms, which resolved within 24 h. This clinical course fulfills the diagnostic criteria [1, 2] of TGA, except for the fact that there was no witness to the onset. This lack did not seem to exclude the diagnosis of TGA because the patient was able to continue driving by herself after the onset, suggesting that her mental status remained unaltered. However, MRI on the next day showed no punctate areas of high intensity on DWIs, which is characteristic of TGA [7]. Instead, the FLAIR images revealed reversible high-intensity areas located in the parietal and occipital subcortical white matter. The reversibility, location, and distribution were characteristic of PRES [4–6]. Furthermore, this patient had a headache, nausea, and high blood pressure. Although each of these symptoms and signs occasionally accompanies TGA [1–3, 8], the constellation of them combined with the neuroimaging findings led us to the diagnosis of PRES

As a case of PRES, this case was atypical both clinically and radiologically. Radiologically, the lesions were small and inconspicuous compared with the typical findings in PRES. In addition, initial imaging was normal. However, severity in radiological findings in PRES is protean, from mild to severe. The mild findings can be as inconspicuous as those in our case [5]. Moreover, the delay in appearance similar to our case has been reported [9]. Furthermore, the lesions in this case had the characteristics of PRES: the multiplicity, reversibility, subcortical location, and parieto-occipital distribution [4–6]. Therefore, although atypical, the radiological findings of this case are consistent with the diagnosis of PRES.

Clinically, this case was unique as it presented like TGA; there have been no reported cases of PRES with a clinical course resembling TGA. Although PRES usually presents with seizures, altered mental status, headache, and visual disturbances, it can present in various ways [4]. Even a case of isolated headache has been reported [10]. Although amnesia has not been described as a common symptom in PRES, two cases of PRES that developed amnesia without altered mental status have been reported [11, 12]. Therefore, presentation with isolated reversible amnesia is consistent with the diagnosis of PRES. In this case, we did not radiologically confirm the involvement of memory-related structure, which raises the possibility of coincidence of both PRES and TGA. However, this lack of radiological involvement does not necessarily indicate the absence of pathological or functional involvement, as in the reported cases with amnesia [11, 12], the involvement of memory-related structures also had not been radiologically demonstrated.

Although TGA is characterized by isolated amnesia, it is accompanied by headache in 7%–40% [1–3] and by nausea in 10%–20% [2, 3]. In addition, it has been reported that many cases of TGA were associated with transiently elevated blood pressure [8]. In our patient, if these features had been attributed to TGA and we had not performed the follow-up MRI, we might have diagnosed the patient merely with TGA. Therefore, similar cases, which may be called “amnesic variant of PRES,” may have been unrecognized.

Our case suggests that PRES can mimic TGA. While TGA is a self-limited disease that resolves within 24 h [1–3], PRES is potentially life-threatening [4]. Therefore, the differential diagnosis is important. PRES should be considered in the differential diagnosis of TGA.
